# Investigation of anti-HIV activity, cytotoxicity and HIV integrase inhibitory activity of polyherbal formulation BH extracts

**DOI:** 10.1186/1471-2334-14-S3-O23

**Published:** 2014-05-27

**Authors:** S Balraj, P Selvam, Y Pommier, M Metifiot, M Christophe, C Pannecouque, E De Clercq

**Affiliations:** 1 Hans Rover Herbal Clinic, Perambalur, Tamil Nadu, India; 2 Nova College of Pharm. Edu and Research, Jupudi, AP, India; 3 Laboratory of Molecular Pharmacology, NCI, Mary Land, USA; 4 Rega Institute for Medical Research, Leuven, Belgium

## Background

HIV integrase (IN) plays important roles at several steps, including viral DNA nuclear import, targeting viral DNA to host chromatin and integration. Identification of novel inhibitors of HIV Integrase has emerged as promising antiviral agents for the treatment of HIV/AIDS. Present work is to investigation of anti-HIV activity and HIV integrase inhibitory activity of various extracts of polyherbal formulation BH.

## Method

Polyherbal extracts (BH) were tested for anti-HIV activity against HIV-1 and -2 in MT-4 cells and cytotoxicity also tested against uninfected MT-4 cells. BH extracts were investigated for inhibition of HIV integrase enzymatic activity to understand the mechanism of antiviral action.

## Results

All extracts exhibited inhibitory activity against HIV-1 integrase (3’P IC_50_: 8.8-63 μg/mL and ST IC_50_: 4.9-65 μg/mL). The ethanolic extract (BH-HT) displayed significant inhibitory activity against both step of HIV in enzymatic activity (3’P IC_50_: 8.8µg/mL and ST IC_50_: 7.5 µg/mL). The ethanolic extract also inhibits the HIV-1 replication at the concentration of 59.30 µg/mL and cytotoxicity was found to be more than >125 µg/mL.

## Conclusion

All the extracts inhibit the HIV integrase activity and ethanolic extract inhibit HIV and Integrase enzyme.

